# Heat shock protein70 is implicated in modulating NF-κB activation in alveolar macrophages of patients with active pulmonary tuberculosis

**DOI:** 10.1038/s41598-017-01405-z

**Published:** 2017-04-27

**Authors:** Chun-Hua Wang, Pai-Chien Chou, Fu-Tsai Chung, Horng-Chyuan Lin, Kuo-Hsiung Huang, Han-Pin Kuo

**Affiliations:** Department of Thoracic Medicine, Chang Gung Memorial Hospital, Taipei, Taiwan

## Abstract

Heat shock proteins (HSPs) have been shown to modulate NF-κB activation. It is unknown whether HSP70 plays a role in modulating NF-κB-mediated pro-inflammatory cytokines released from alveolar macrophage (AM) of patients with active pulmonary tuberculosis (TB). Peripheral blood monocytes (PBMs) and AM were sampled from nineteen active TB patients and 14 healthy individuals. HSP70 expression was 3-fold higher in AMs of active TB patients than normal subjects, and declined after receiving 3-month anti-TB treatment. Overexpression of HSP70 by transfection with HSP70 plasmid decreased p-IκBα and p65 NF-κB activities. Inhibition of NF-κB activation using NF-κB or MAPK inhibitors increased HSP70 expression in AM of TB patients. Blocking p38- or ERK-MAPK decreased NF-κB and IκB activities, leading to up-regulated HSP70 expression. Overexpression of HSP70 alone or with p38 or ERK inhibitors decreased TNF-α (57%, 83% and 74%, respectively) and IL-6 (53%, 70%, and 67%, respectively) release from macrophages of TB patients. In conclusion, HSP70 modulates NF-κB activation in AM of TB patients, through inhibiting IκB-α phosphorylation or acting as a chaperon molecule to prevent NF-κB binding to the target genes by facilitating degradation. The upregulated HSP70 may suppress the release of pro-inflammatory cytokines during active PTB infection, and prevent overwhelming tissue damage.

## Introduction

Tuberculosis (TB) remains a major health problem worldwide^1^. Clinical and pathologic features of TB depend at least in part on the orchestrated secretion of a number of pro-inflammatory cytokines, such as tumor necrosis factor (TNF)-α, interleukin (IL)-1β and IL-6. The transcription factor NF-κB regulates gene expression in response to various extracellular stimuli, including TNF-α, IL-1β and lipopolysaccharide^2, 3^. Our previous report demonstrated that alveolar macrophages (AM)^4^ or monocytes^5^ from patients with active pulmonary TB may release pro-inflammatory cytokines TNF-α and IL-1β via NF-κB activation. The inflammatory responses in turn effectively eliminated the proliferation of TB bacilli by up-regulating phagocytic capacity and cytotoxicity of macrophages^6^, and thus limiting further mycobacterial growth by inducing granulation formation^7, 8^.

Heat shock proteins (HSPs) are a group of stress proteins that mediate cellular and tissue protection against diverse cytotoxic stimuli^9^ and act as key regulators of the host’s immune system^10^. HSP70 delivers peptide antigen to human DCs and stimulates them to generate effective T-cell functional responses^11^. In mycobacterial infection, pathogen recognition by toll-like receptors (TLRs) and downstream TLR signaling play an important role in activation of innate immune cells and prevent excessive T cell-mediated inflammation^12^. Upon stimulation with TLR4 ligand such as LPS downstream TLR/MyD88-dependent signaling results in activation of NF-κB-mediated transcription of pro-inflammatory cytokines such as TNF-α and IL-6^13^. Members of the HSP family may cross-talk with toll-like receptors to activate pro-inflammatory signals^14^, and play an important role in granuloma formation and immune protection during *M. tuberculosis* infection. HSP70 may block the activation of NF-κB^2, 15, 16^, and inhibit cytokine-mediated NF-κB nuclear translocation and subsequent pro-inflammatory cytokine release^17^.

We have conducted a prospective study to investigate the role of HSP70 in suppressing NF-κB-mediated TNF-α and IL-6 release. We also have explored the interaction between HSP70 and NF-κB by over-expression of HSP70 or inhibition of NF-κB activation, or blocking the activity of mitogen-activated protein kinases (MAPKs) that are required for persistent NF-κB activation^18^.

## Results

### Cell profiles in BAL

Table 1 summarizes the BAL findings on the TB patients and control subjects. The recovery rate of BAL was significantly lower in patients with active pulmonary TB than in the control subjects. There was a significant increase in total cell counts in patients with active pulmonary TB (38.6 ± 7.4 × 10^6^ cells, n = 19) compared to those of the control subjects (8.2 ± 0.7 × 10^6^ cells, n = 14, p < 0.001). The proportions of lymphocytes and neutrophils were significantly higher in patients with TB (8.7 ± 2.4% and 13.3 ± 4.5%, n = 19, respectively) than in the control subjects (2.6 ± 1.0% and 1.0 ± 0.2%, n = 14, p < 0.05, respectively). Reciprocally, the percentage of alveolar macrophages (AM) was significantly lower in patients with TB (79.0 ± 5.0%, n = 19) than in the control subjects (96.1 ± 1.0%, n = 14, p < 0.05).

**Table 1:** Characteristics of bronchoalveolar lavage in control subjects and patients with active pulmonary tuberculosis.

	Tuberculosis (n = 19)	Control subjects (n = 14)
Total cell count, ×10^6^	38.6 ± 7.4**	8.2 ± 0.7
Recovered volume, %	43.8 ± 4.0	62.4 ± 3.1
Cell viability, %	93.7 ± 0.8	94.7 ± 0.8
AM, %	79.0 ± 4.5*	96.1 ± 1.0
Lymphocytes, %	8.7 ± 2.4*	2.6 ± 1.0
Neutrophils, %	13.3 ± 4.5*	1.0 ± 0.2

AM = alveolar macrophages. Values presented as means ± SEM.

*p < 0.05, **p < 0.001 compared with control subjects.

### Expression of HSP70 in PBM and AM before and after treatment for PTB

**Figure 1:**
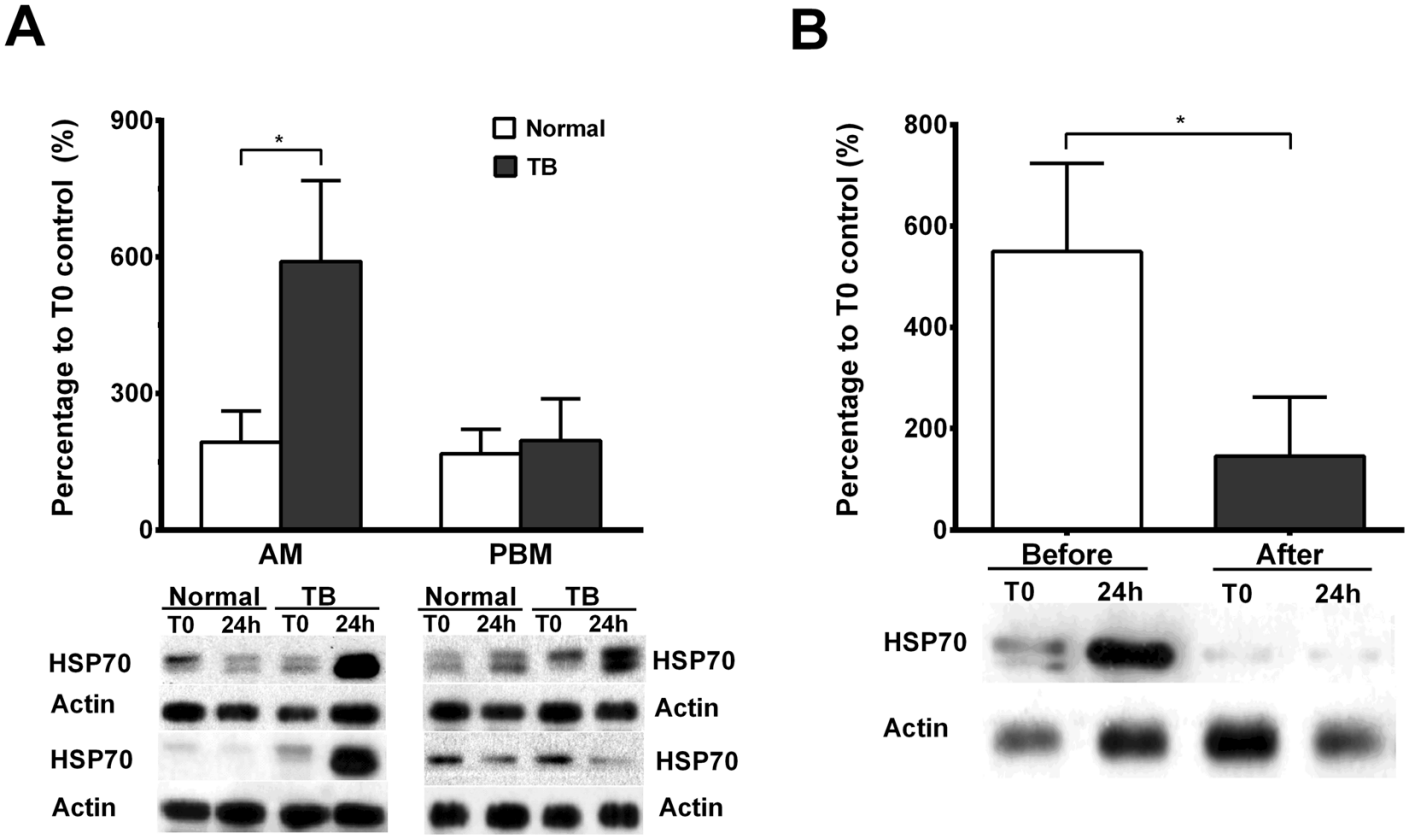
(**A**) Levels of HSP70 in alveolar macrophages (AM) and peripheral blood monocytes (PBM) quantified by western blot analysis in patients with active pulmonary TB (TB, n = 6) and normal subjects (Normal, n = 6). *p < 0.05 compared with normal subjects. (**B**) Levels of HSP70 in AM from TB patients (n = 5) before and after receiving anti-TB treatment for 3 M quantified by western blot. *p < 0.05 compared with before anti-TB treatment.

Western blot analysis revealed higher expression of HSP70 in alveolar macrophages of TB patients compared to normal subjects by a 3-fold difference (n = 6, p < 0.05). However, the expression of HSP70 in the peripheral blood monocytes (PBM) was not significantly different between TB patients and normal subjects (Fig. 1A). The expression of HSP70 in AM of TB patients significantly decreased from 549.6 ± 173.9% (percentage to control) to 146.3 ± 115.8% (percentage to control) three months after standard anti-TB treatment (n = 5, p < 0.05) (Fig. 1B).

### Interaction between HSP70 and NF-κB activity

**Figure 2:**
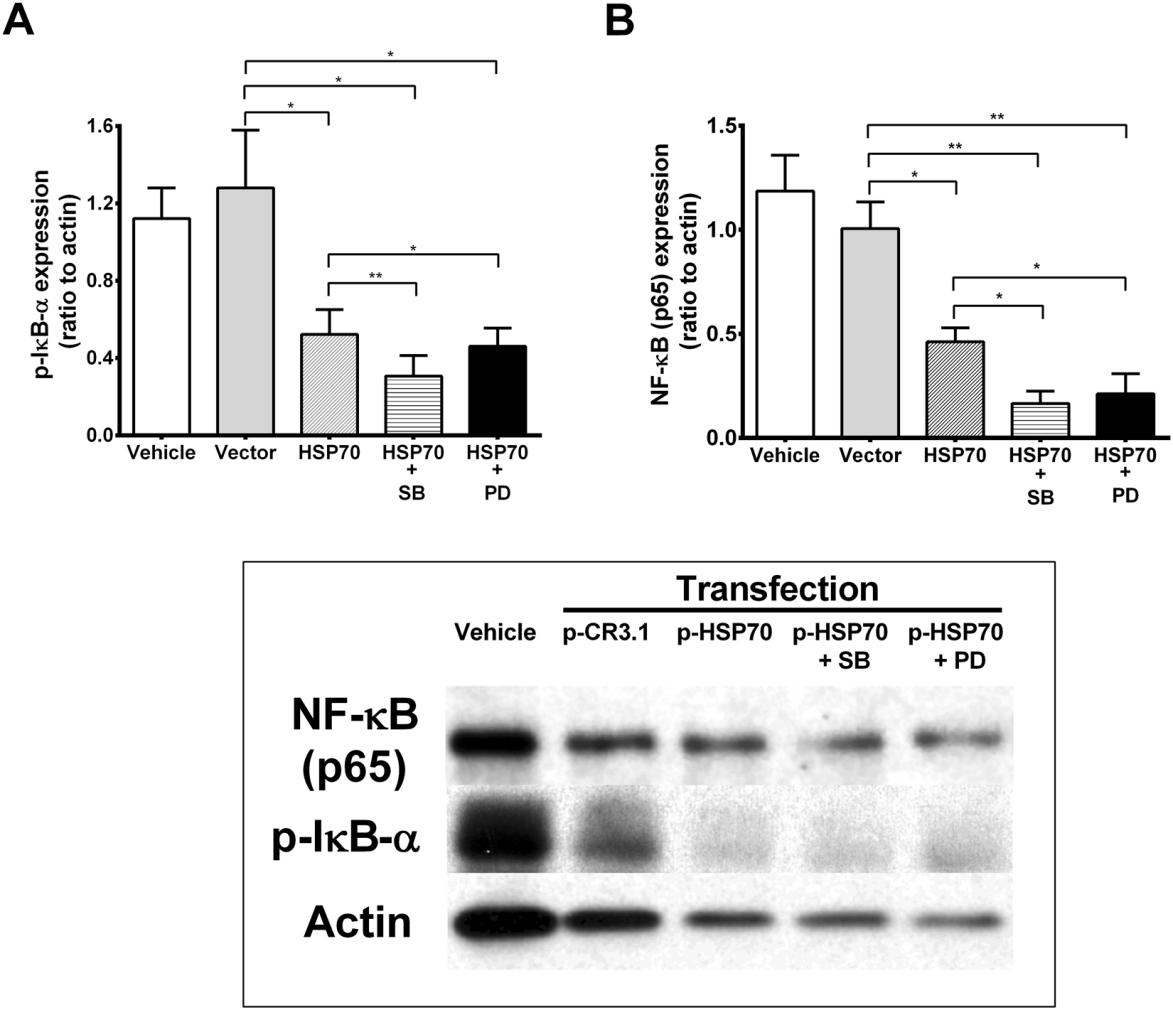
Western blot analysis to quantify the expression of IκB-α (**A**) and NF-κB p65 activation (**B**) in HSP70 overexpression (HSP70) of alveolar macrophages derived from TB patients (n = 5) in the absence or presence of a p38 kinase (SB) or ERK kinase inhibitor (PD). **p < 0.01, *p < 0.05 compared with vector transfection control (Vector).

**Figure 3:**
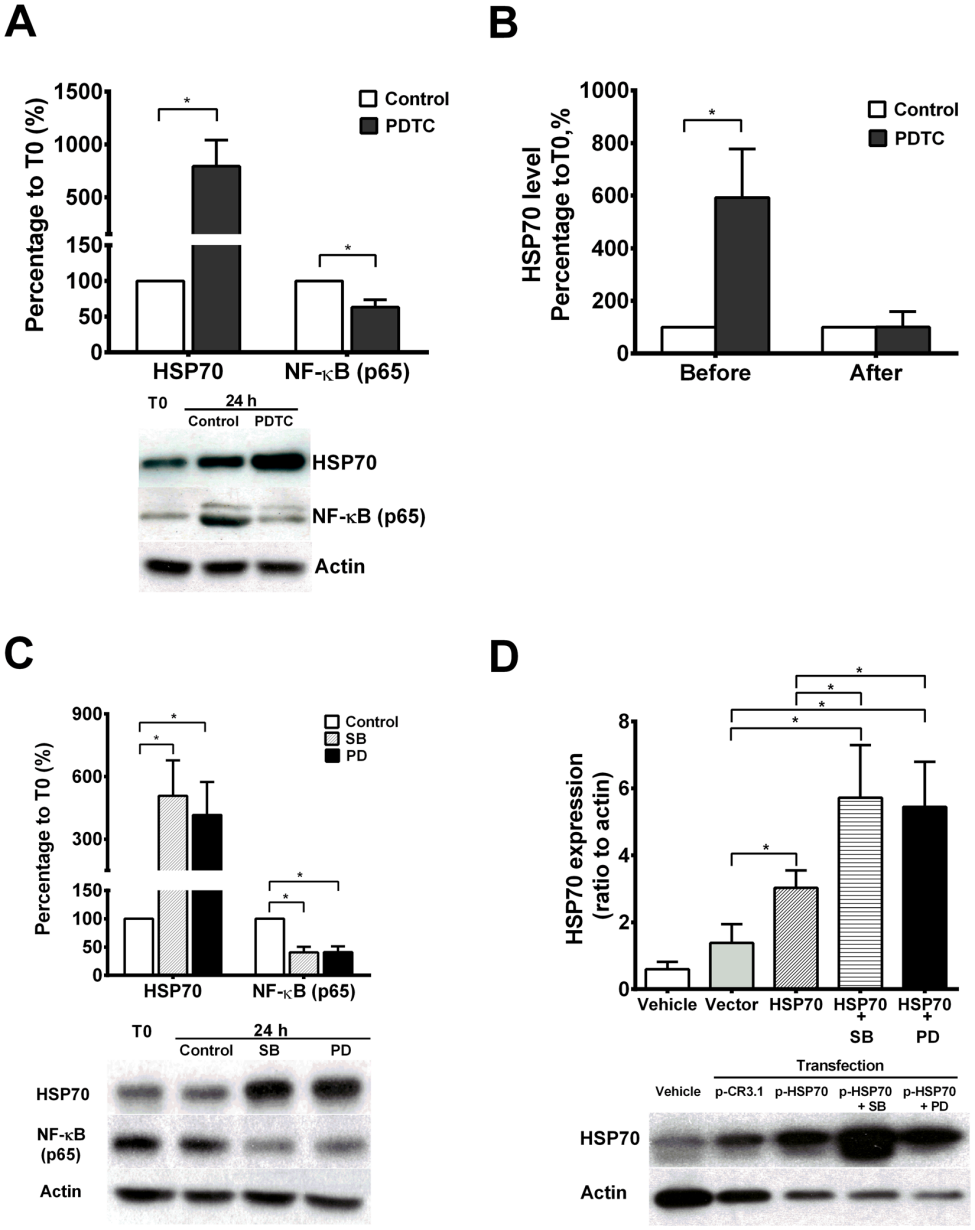
(**A**) Levels of HSP70 and NF-kB p65 in alveolar macrophages of TB patients (n = 6) quantified by western blot analysis in the absence or presence of a NF-kB inhibitor (PDTC). *p < 0.05 compared with control. (**B**) HSP70 levels in AM from TB patients (n = 4) quantified by western blot analysis in the absence or presence of a NF-kB inhibitor (PDTC) before or after anti-TB treatment for 3 M. *p < 0.05 compared with control. (**C**) Western blot analysis to quantify the expression of HSP70 and NF-κB p65 in AM from TB patients (n = 7) in the absence or presence of a p38 kinase (SB) or ERK kinase inhibitor (PD). *p < 0.05 compared with control. (**D**) Levels of HSP70 in HSP70 overexpressed AM (HSP70) from TB patients (n = 5) in the absence or presence of a p38 kinase (SB) or ERK kinase inhibitor (PD). *p < 0.05 compared with vector transfection control (Vector).

To investigate the role of HSP70 in AM of active TB, AM of TB patients were transfected with HSP70 plasmid. Overexpression of HSP70 significantly decreased IκB-α phosphorylation (p-IκB-α) and led to a decrease in NF-κB activation (Fig. 2A,B). However, inhibition of NF-κB activation by pyrrolidine dithiocarbamate (PDTC), an NF-κB inhibitor, significantly enhanced the expression of HSP70 in AM of TB patients (Fig. 3A). There was no further enhancement of HSP70 expression by NF-κB inhibitors in AM of TB patients 3 months after anti-TB treatment (Fig. 3B).

**Figure 4:**
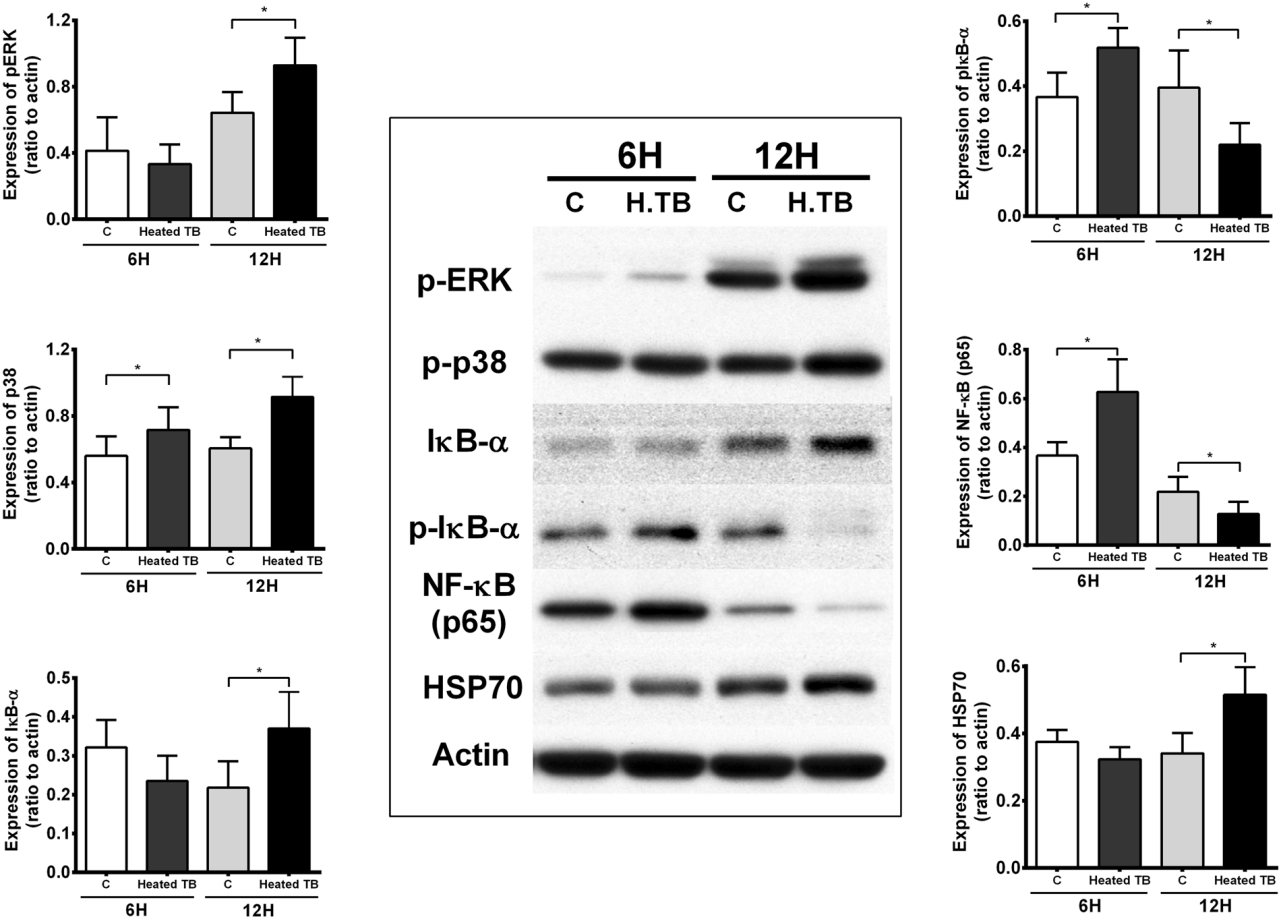
Western blot analysis to quantify the expression of MAPK (p38 and ERK), IκB-α, NF-κB p65 and HSP70 in alveolar macrophages of normal subjects (n = 6) after incubation with heated TB bacilli for 6 and 12 hours. *p < 0.05 compared with control.

To delineate the causal effect of NF-κB activation on HSP70 expression, the upstream signaling pathway for TB induced NF-κB activation was explored. AM from normal subjects (n = 6) increased phosphorylation of MAPK (p38), IκB-α and NF-κB at 6 h of incubation with heated TB bacilli (5 μg/ml) (Fig. 4). After 12 h of incubation, there was an increase in HSP70 expression and a concomitant decrease in IκB-α phosphorylation, IκB-α degradation and NF-κB (p65) activity, despite of a persistent enhancement of p38 and ERK activation (Fig. 4). Inhibition of MAPK either with SB20358, a p38-MAPK inhibitor, or PD98059, an ERK inhibitor, significantly suppressed NF-κB activation with a concomitant increase in the HSP70 expression in AM of TB patients (Fig. 3C). Even in HSP70-overexpressed AM of TB patients, treatment with SB20358 or PD98059 further increased HSP70 expression (by 3.1-fold and by 2.9-fold, respectively) (Fig. 3D).

### Generation of pro-inflammatory cytokines

**Figure 5:**
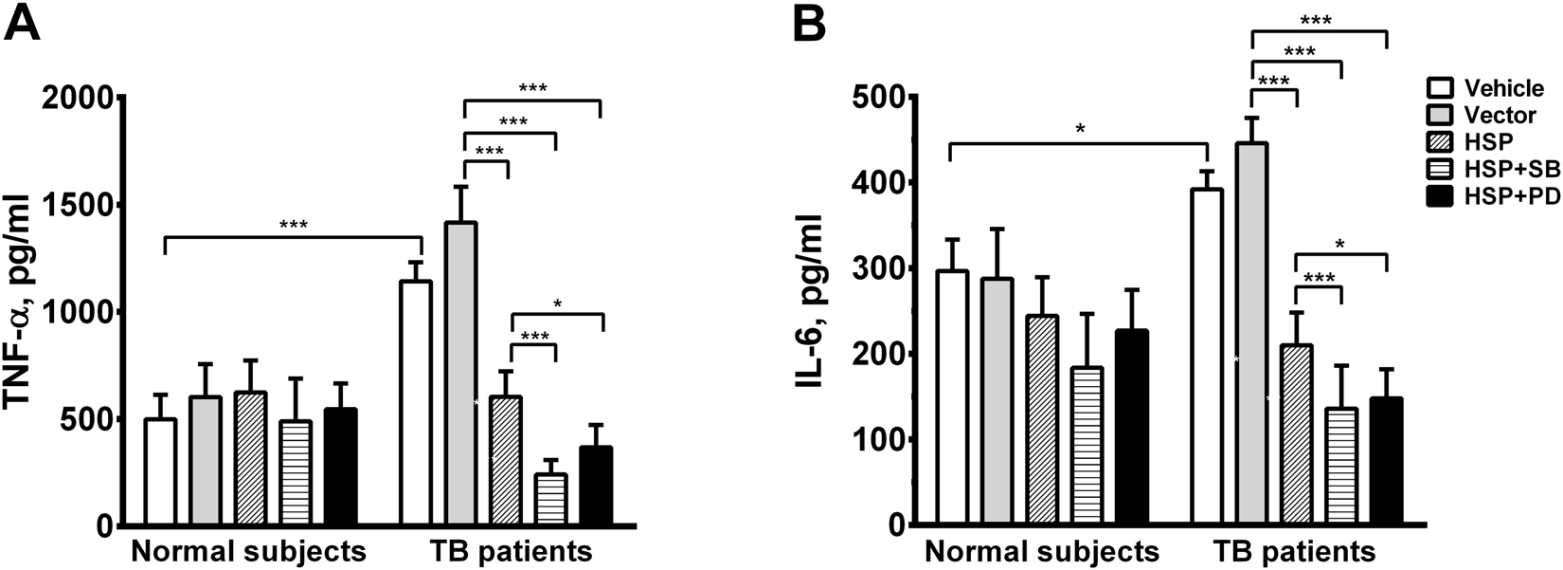
Levels of TNF-α (**A**) or IL-6 (**B**) secreted from AM of TB patients (n = 10) and normal subjects (n = 7) incubated in culture medium (Vehicle), or transfected with vector alone (Vector), or transfected with HSP70 plasmid (HSP) in the absence or presence of a p38 kinase (SB) or ERK kinase inhibitor (PD). ***p < 0.001, **p < 0.01, *p < 0.05.

Cultured AM from the TB patients released higher levels of TNF-α (1143.0 ± 88.2 pg/ml, n = 10, p < 0.001) (Fig. 5A) and IL-6 (392.1 ± 21.2 pg/ml, n = 10, p < 0.05) (Fig. 5B) into the culture medium than those of the normal subjects (499.4 ± 114.5 pg/ml and 296.6 ± 36.6 pg/ml, respectively, n = 7). Transfection with HSP70 in AM of TB patients to overexpress HSP70 (increased by 1.9-fold expression) significantly inhibited basal generation of TNF-α (603.7 ± 119.3 pg/ml, n = 10, p < 0.01) (Fig. 5A) by 57.4%, and IL-6 (209.8 ± 38.4 pg/ml, n = 10, p < 0.01) by 52.9%, but not normal subjects (Fig. 5B). Treatment with SB20358 or PD98059 further decreased the release of TNF-α (by 83.0% or 74.0%, respectively) or IL-6 (by 69.5% or 66.7%, respectively) from AM of TB patients (Fig. 5A,B). However, there was no significant effect of transfection of HSP70 in the presence or absence of MAP kinase inhibitor on cytokine production from AM of control subjects (Fig. 5A,B).

## Discussion

Our present study has demonstrated that HSP70 expression is upregulated in AM of patients with active TB and inhibits NF-κB-mediated TNF-α and IL-6 release. The upregulated expression of HSP70 in AM decreased after effective anti-TB treatment, and was not seen in the peripheral blood monocytes, suggesting HSP70 upregulation is attributed to a direct exposure of AM to *Mycobacterium TB*. The inhibition of NF-κB activity indicates HSP70 plays a role in modulating the inflammatory responses during the process of pulmonary TB infection.

Transcription of most pro-inflammatory cytokine genes is regulated by NF-κB activation. Acting as a ubiquitous transcription factor, NF-κB plays an essential role in the regulation of a variety of genes involved in immune functioning, inflammatory response, endothelial cell activation, and the control of cell growth^19, 20^. Our previous reports and the present study have demonstrated that the activities of NF-κB in AM are upregulated and contribute to the increased release of pro-inflammatory cytokines in patients with active pulmonary TB^4^. Exposure to Mycobacterium TB activates the mitogen-activated protein kinases (MAPKs) family through toll-like receptor in of AM^21^, and through signaling lymphocyte activation molecule in IFN-γ-producing T cells^22^. The latter is directly correlated with the responsiveness to *M. tuberculosis* antigen and contributes to MAPK activation mediated IFN-γ production. In this study, we have shown NF-κB activity was up-regulated in AM of TB patients and was suppressed by the treatment with ERK or p38 MAPK inhibitors, suggesting ERK and p38 signaling pathways are essential in NF-κB activation in AM of TB patients. NF-κB activation requires activation of IκB kinase (IKK) to phosphorylate IκB-α and release p65/p50 dimers that migrate into the nucleus and bind to NF-κB binding sites of several pro-inflammatory gene promoter sites. In this study, the up-regulation of HSP70 in AMs incubated with heated TB bacilli was associated with a concomitant decrease in IκB-α phosphorylation, IκB-α degradation and NF-κB activity, despite persistent activation of p38 and ERK MAPK. This result may suggest that up-regulation of HSP70 leads to the inhibition of IκB-α phosphorylation which in turn suppresses NF-κB activation. These results were further confirmed by the observation that overexpression of HSP70 in AM of TB patients inhibited IκB-α phosphorylation and NF-κB activity. HSP70 has been shown to stabilize IκB-α through the prevention of IκB kinase (IKK) activation and thereby inhibit NF-κB activation^23^. HSP70 was also shown to inhibit IκB-α phosphorylation by inhibiting IκB-α degradation^24^. Therefore, further studies with immunoprecipitation assays or with immune complex kinase assay are needed to demonstrate HSP70 inhibition of IκB-α ubiquitination or to demonstrate a direct inhibition of IκB kinase by HSP70, respectively in AM of TB patients. Furthermore, an inhibition of MAPK, either p38 or ERK, increased HSP70 expression may suggest that it is also possible that HSP70 may directly interact with MAPK to modulate IKK activation and then NF-κB activity. HSP70 has been suggested to be related to the phosphorylation of MK2, a specific nuclear downstream target of MAPK p38, indicating HSP70 is a potential chaperone for the nuclear translocation of p38^25^.

In this study, MAPK activity was investigated to demonstrate the rationale for modulation of NF-κB activation by an inhibition of upstream signaling pathway to further explore the causal effect of NF-κB activation on HSP70 expression. Since AM or PBMC from active TB patients may have been stimulated by mycobacteria and a variety of mycobacteria infection-derived pro-inflammatory mediators, the expression of MAPK (p38 and ERK), IκB-α, NF-κB p65 and HSP70 may not represent the responses to mycobacteria exposure alone. We therefore observed the time-course change of NF-κB signaling and HSP70 expression in AM of normal subjects after exposure to TB bacilli *in vitro* instead of AM of active TB patients.

HSP also assists in the delivery of target proteins to the ubiquitin-proteosome system for degradation through co-chaperone molecules^26^. HSP70 has been shown to translocate to the nucleus and associates with the nuclear PDZ-LIM domain protein PDLIM2 acting as a ubiquitin E3 ligase that targets the p65 subunit of NF-κB for degradation^27^. Thus, the up-regulation of HSP70 in AM of TB patients may facilitate degradation of both NF-κB and HSP70 proteins. This is supported by the observation that an inhibition of NF-κB by either p38 or ERK MAPK inhibitors or a NF-κB inhibitor (PDTC) increased nuclear HSP70 expression, i.e the less NF-κB activation, the less NF-κB translocation to the nucleus, the less HSP70 required for NF-κB degradation, leading finally to higher levels of HSP70.

Furthermore, HSP70 overexpression either by transfection of HSP70 plasmid or by MAPK inhibitors attenuated NF-κB-mediated TNF-α and IL-6 release from AM of TB patients. Ribeiro *et al*.^28^ also demonstrated the HSP70 bound to intracellular TNF-α prevented its release from endotoxin-treated alveolar macrophages. The up-regulated HSP70 may also act as a chaperone molecule bound to TNF-α and IL-6, and prevent their release. Nevertheless, HSP70 is implicated in preventing excessive inflammatory responses of pulmonary TB infection, and thereby can protect against cell injury by these diverse pathophysiological stresses.

Limitation of the study is that the relatively few patients were recruited into this study. Considering the risk of bronchoscopy, several patients with pulmonary TB declined to undertake the bronchoalveolar lavage study. A larger number of participants would have provided the opportunity to further investigate the detailed mechanisms underlying HSP70 up-regulation in pulmonary TB, and the interactions between HSP70 and NF-κB activation. Although it is well known that p38- and ERK-MAPK in AM or PMB are upregulated upon mycobacterial infection or mycobacterial components, it is essential to delineate the precise mechanisms underlying the inhibitory effects of HSP70 on NF-κB activity by investigating the basal expression of MAPK before and after HSP70 overexpression in AM or peripheral monocytes before and after anti-TB treatment. We recognize the importance of these studies in exploring the underlying mechanisms of HSP70, and will carry out in the near future.

In conclusion, our study indicates the role of HSP70 in patients with active pulmonary TB, and shows that HSP70 levels decline with resolution of the inflammatory responses after successful treatment of TB. We have demonstrated that HSP70 plays a role in modulation of NF-κB activation in alveolar macrophages of TB patients, probably through inhibiting IκB-α phosphorylation and IκB-α degradation or acting as a chaperone molecule to prevent NF-κB p65 subunit binding to the target genes by facilitating degradation. The upregulated HSP70 may suppress the release of pro-inflammatory cytokines during active pulmonary TB infection, and prevent overwhelming tissue damage.

## Methods

### Patient Population

Nineteen patients with proven active pulmonary TB (PTB) (13 men and 6 women, aged 59.1 ± 4.5 years) were studied before anti-TB treatment. None of these patients were current smokers. For all patients, chest radiographs were performed for the diagnosis of PTB, and at least one recent sputum specimen was positive for acid-fast bacilli on microscopic examination. Sputum culture revealed *M. tuberculosis*. Patients with chronic airway diseases such as asthma, chronic obstructive pulmonary disease, bronchiectasis, pneumonia, malignancy, or patients with systemic inflammatory diseases, such as lupus erythromatosus, sepsis, or HIV infection were excluded from this study. None of them had poor nutritional status assessed by triceps skinfold thickness, midarm circumference and serum albumin. None took oral corticosteroids or other immunosuppressants within 3 months before study.

Fourteen healthy subjects including 8 men and 6 women were recruited, with a mean age of 47.3 ± 4.3 years. None of these subjects had a history or evidence of lung disease based on physical and chest radiographic examinations. All subjects were nonsmokers. None of them had any upper respiratory tract infection within the recent 6 weeks. None of them took any antibiotic or medication during the evaluation. Ethical approval was obtained from the Institutional Review Board of Chang Gung Memorial Hospital. All experiments were carried out in accordance with the relevant guidelines and regulations. Informed consents were written and obtained from all subjects prior to inclusion in the study.

### Preparation and culture of peripheral blood monocytes (PBM)

Thirty ml heparinized blood were collected from normal subjects and patients with active PTB. Peripheral mononuclear cells were isolated on a Ficoll-Hypaque (Sigma, St Louis, MO, USA) density gradient (*d* = 1.078 g/cm^3^), and were washed three times in a large amount of RPMI-1640 to decrease the attachment of platelets. The mononuclear cells were then resuspended (10^6^ cells/ml) in RPMI-1640 (GIBCO, Grand Island, NY, USA) medium containing 5% fetal calf serum (FCS), 100 U/ml penicillin, and 100 μg/ml streptomycin. PBMs were purified from these peripheral mononuclear cells by the adherence method. The purity of monocytes was >90% recognized by morphologic identification by modified Wright-Giemsa stain and nonspecific esterase stains exclusion.

### Preparation of lower respiratory tract cells

Bronchoalveolar lavage (BAL) was performed in all study subjects using five aliquots (50 ml each) of 0.9% saline solution as described previously^4, 29^. Briefly, sterile saline solution was introduced into the right fourth or fifth subsegmental bronchus in normal subjects. In TB patients, BAL was performed from the involved bronchi. The lavaged fluid was retrieved by gentle aspiration, then pooled and filtered through two layers of sterile gauze to avoid contamination. Thereafter, BAL fluid was centrifuged at 600 g for 20 min at 4 °C. The cell pellet was washed sequentially and resuspended in RPMI-1640 (GIBCO, Grand Island, NY, USA) supplemented with 5% heat-inactivated fetal calf serum (FCS, Flow Laboratories, Paisley, Scotland, UK) at 10^6^ cells per ml. The cell viability was determined by trypan blue exclusion. Differential cell counts were determined by counting 500 cells on cytocentrifuge preparations using a modified Wright-Giemsa stain.

Alveolar macrophages (AM) was purified as previously described^4^, AM were placed in plastic culture dishes in RPMI-1640, allowed to adherence for 60 min and washed for three times with warm RPM-1640 to remove non-adherent cells. Adherent cells were scraped off with a sterile rubber policeman. Such detachment method retrieved >96% purified AM. After adjustment of cell viability, the cells were resuspended (10^6^ cells/ml) in RPMI-1640 medium containing 5% FCS, 100 U/ml penicillin, and 100 μg/ml streptomycin.

### Study protocol

The purified AM or monocytes were placed in 12 well petri dishes at 10^6^ cells/ml for 24 h at 37 °C, 5% CO_2_. The culture supernatant was collected and frozen at −70 °C until assay. The cytosol proteins of AM/monocytes from patients with active PTB or normal subjects were extracted to elucidate the expression of HSP70 at baseline. To examine the effect of NF-κB, HSP70 expression and ERK or p38-MAP kinase activity, AM from patients with active PTB or normal subjects were cultured at baseline or after various treatments. To examined the effect of HSP70 expression on NF-κB activation and ERK or p38-MAP kinase activity, AM from patients with active pulmonary TB or normal subjects were transfected with plasmid containing human HSP70 cDNA, then cultured for 24 h at 37 °C, 5% CO_2_. To clarify which MAP kinase signaling pathway was implicated in the HSP70 expression, AM either alone or with human HSP70 cDNA transfection was incubated with an inhibitor (30 μM) for 24 h at 37 °C, 5% CO_2_. The supernatants were stored at −70 °C until assay for cytokines.

To clarify the involvement of MAPK and HSP70 in NF-κB and phosphorylated IκB-α activity, the purified AM from normal subjects (n = 6) in 12 well petri dishes at 10^6^ cells/ml was incubated with heated TB bacilli (Sigma, St Louis, MO, USA) in presence or absence of 5 μg/ml for 6 and 12 h at 37 °C, 5% CO_2_. The expression of NF-κB p65 and HSP70, p-ERK, p-p38, IκB-α and p-IκB-α in 4 × 10^6^ cells/ml purified AM were studied by Western immunoblot.

## Cloning of human heat shock protein (HSP) 70 gene and construction with human HSP 70 cDNA

Total cellular RNA from human AMs was harvested by the acid guanidium-thiocyanate phenol-chloroform method as recommended by the manufacturer (Promega, Madison, WI). After reverse transcription (50°, 1 hr), the human HSP70 coding region was amplified by polymerase chain reaction (PCR) from the HSP70 cDNA, using the following two oligonucleotide primers: 5′-AGGGAACCGCCATGGCCAAA-3′ (Sense); 5′-TCTTGGAAAGGCCCTCA-3′ (Anti-sense). The 5′-sense primer was containing the consensus Kozak sequence fused to HSP70 coding N-terminal amino sequences. The 3′ anti-sense primer contained HSP70 coding C-terminal amino sequences downstream of stop codon. Cycling condition was 94 °C for 1 minute, 45 °C for 1 minute, and 72 °C for 2 minutes, followed by a final cycle at 72 °C for 5 minutes. The PCR products were isolated by agarose gel electrophoresis. Automated sequencing was performed using either Sequenase or Tag double-stranded dideoxy terminator kit (Applied Biosystems, Inc, Foster City, CA) as recommended by manufacturer. Sequences were analyzed using the Genetics Computer Group software (Madison, WI). Databases were searched using the BLAST algorithm. The fragment containing the open reading frame (ORF) of the cloned human HSP70 cDNA was inserted in frame into the A-T ligation site of the pCR3.1 plasmid (Promega, CA) and further ligased into the Xho I and EcoRI sites of the pEGFP-C2 plasmid (Clontech, Palo Alto, CA). The Orientation of the inserts was determined by restriction enzyme mapping and sequencing. The resulting pCR3.1-HSP70 plasmids were further sequenced for confirmation of the in-frame fusion of HSP70 to the CMV promoter and EGFP cDNA 5′ to the HSP70 open reading frame.

### *In vitro* transfection with pCR3.1-HSP 70 plasmid

Purified AMs from TB patients or normal subjects were washed three times with pre-warmed Opti-MEM. The method of transient transfection was described previously^4^. Briefly, HSP70 antisense or sense oligonucleotides or pCR3.1-HSP70 was premixed with 10 mg/ml Lipofectin reagent in Opti-MEM at the desired concentration at room temperature for 15 min, then diluted 5-fold with Opti-MEM. 500 ml of diluted Lipofectin complex were added to AM. Transfections were carried out for 6 hours, after which the medium was removed and replaced with RPMI-1640 containing 10% FCS. Then, the cells were incubated in the presence or absence of SB20358, a p38-MKAP inhibitor (10 mM), or PD98059, an ERK inhibitor (30 mM) for 24 h at 37 °C, 5% CO_2_.

### Preparation of cytoplasmic and nuclear extracts

Cytoplasmic and nuclear extracts were prepared from 4 × 10^6^ monocytes or AM. Briefly, cells were pelleted in microfuge tubes and resuspended in 400 μl of buffer A (10 mM HEPES, pH 7.9: 10 mM KCl; 0.1 mM EDTA, pH 8.0; 0.1 mM EGTA, pH 7.8; 1 mM DTT; 0.5 mM PMSF; 5 mg/ml pepstatin A; 5 mg/ml leupeptin; 5 mg/ml antipain; 100 mg/ml chymostatin; and 5 mg/ml aprotinin) and left on ice for 15 min. Then, 25 μl of Nonidet P-40 (Fluka) was added, and the cells were agitated vigorously. After centrifugation for 30 s, supernatants were aspirated for immunoblot analysis. The pellet containing the nuclear extract was dissolved in 50 μl of buffer C (20 mM HEPES, pH 7.9; 0.4 M NaCl; 1 mM EDTA; 1 mM EGTA; 1 mM DTT; 1 mM PMSF; 5 mg/ml pepstatin A5; 5 mg/ml leupeptin; 5 mg/ml antipain; 100 mg/ml chymostatin; and 5 mg/ml aprotinin). The tube was then vigorously rocked at 4 °C for 15 min on a shaking platform. Protein concentrations in the nuclear extracts were determined by the BioRad method (Hercules, CA, USA).

### Western blot analysis

Aliquots containing 30 µg of total protein were resolved on 10% SDS-PAGE, and transferred to nitrocellulose. The membranes were blocked with 5% skim milk-PBS/0.1% Tween 20 for 1 h before overnight incubation at room temperature with mouse monoclonal anti-HSP70, mouse anti-human NF-κB p65 subunit, and diluted 1:1000 in 5% skim milk-PBS/0.1% Tween 20. Blots were developed by addition of peroxidase substrate with enhancement by ECL (Amersham, Arlington, Heights, IL, USA) solution, and then exposed to XAR films (Eastman Kodak, Rochester, NY, USA).

### TNF-α and IL-6 Assay

The samples were obtained from the supernatant of cultured or stimulated AM culture and were stored at −72 °C before measurement. Total TNF-α and IL-6 levels in all samples were measured using a commercial “sandwich” Enzyme Amplified Sensitivity Immunoassay (PerSeptive Diagnostics, Inc., Cambridge, MA, USA) according to the protocols. Absorbance was read at 450 nm.

### Statistical analysis

Data are presented as mean  SE. One-way ANOVA (analysis of variance) for mixed design was used to compare hemodynamic values of more than two different experimental groups. If variance among groups was noted, a Bonferroni test was used to determine any significant difference between specific points within groups. The data were analyzed by Student’s t-test for paired or unpaired data. For data with even or uneven variation, a Mann-Whitney U test or Wilcoxon signed rank test was used for unpaired or paired data, respectively. A *p* value less than 0.05 was considered significant.

## Electronic supplementary material

Supplementary Information
