# Supplementary material for: Heat shock protein70 is implicated in modulating NF-κB activation in alveolar macrophages of patients with active pulmonary tuberculosis
Source: Sci Rep. 2017 Apr 27;7:1214. doi: 10.1038/s41598-017-01405-z (PMC5430681; doi:10.1038/s41598-017-01405-z)
Supplement: Supplementary file 1 — Supplementary Information [file 41598_2017_1405_MOESM1_ESM.pdf]

Supplementary Information

Heat shock protein70 is implicated in modulating NF-κB activation in alveolar macrophages of patients with active pulmonary tuberculosis

Chun-Hua Wang, Pai-Chien Chou, Fu-Tsai Chung, Horng-Chyuan Lin, Kuo-Hsiung Huang, Han-Pin Kuo

Department of Thoracic Medicine, Chang Gung Memorial Hospital, Taipei, Taiwan

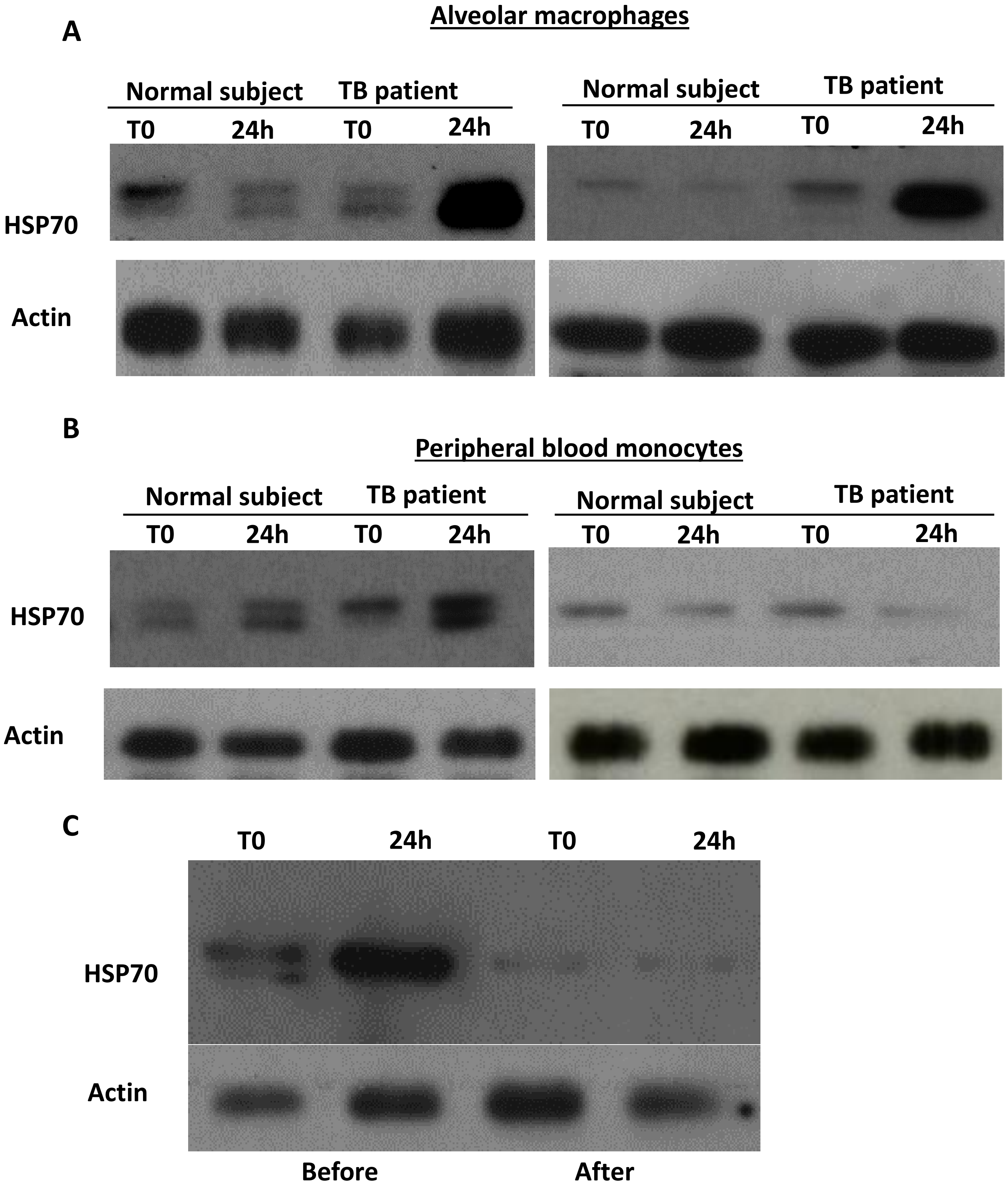

Supplementary Figure 1. Expression of HSP70 in alveolar macrophages or peripheral blood monocytes. The lysate of alveolar macrophages and peripheral blood monocytes derived from normal subject or TB patient was collected at baseline (T0) and after 24-hour culture (24h), and determined by immunoblot analysis of HSP70 and actin by using specific antibodies. The unprocessed immunoblots of (A) alveolar macrophages and (B) peripheral blood monocytes were listed. (C) Immunoblots of alveolar macrophages from TB patient before and after receiving anti-TB treatment for 3 months.

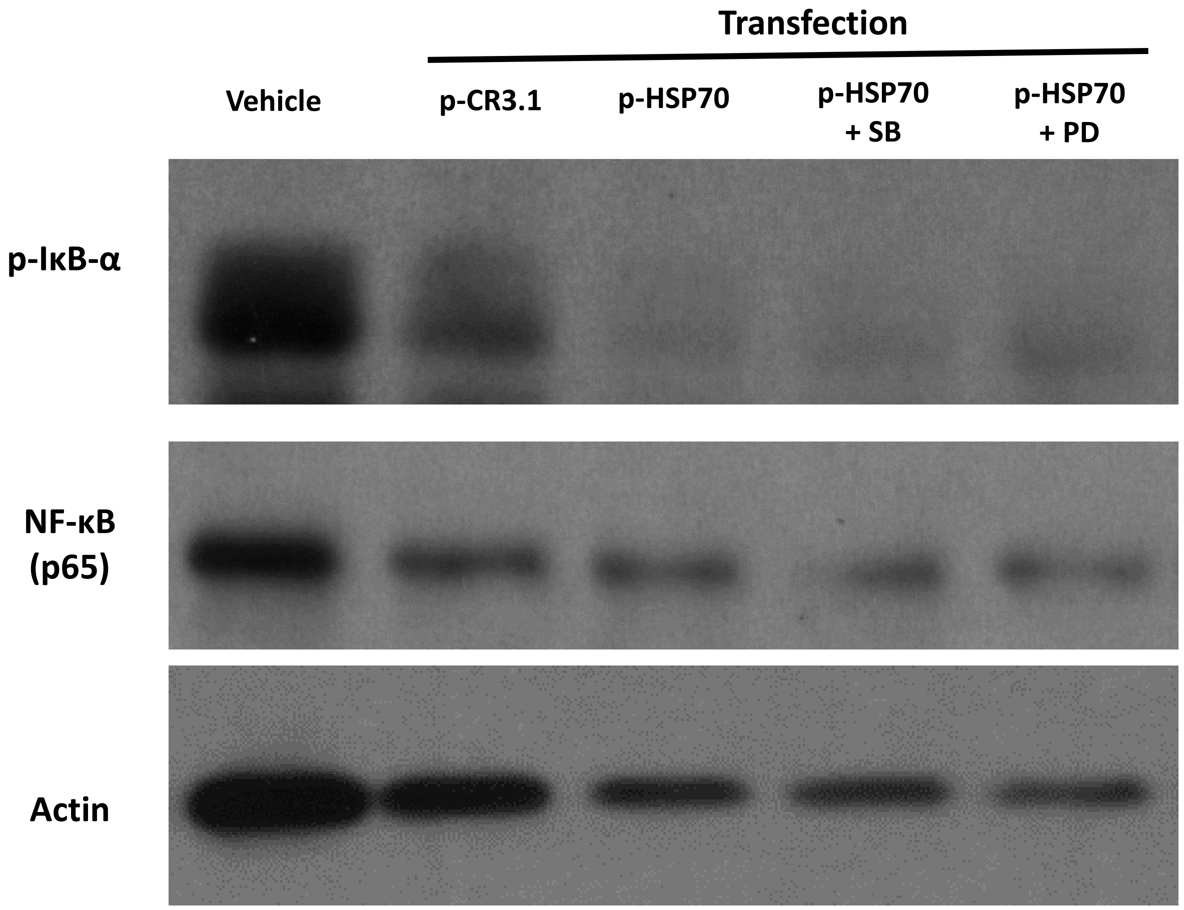

Supplementary Figure 2. Immunoblots of alveolar macrophages by HSP70 overexpression. Alveolar macrophages derived from TB patient were transfected with p-CR3.1 vector or p-CR3.1-HSP70 plasmid (p-HSP70), then incubated with medium alone (vehicle), in the absence or presence of a p38-MKAP inhibitor (p-HSP70+SB) and an ERK inhibitor (p-HSP70+PD) for 24 hours. The lysate of these cells was collected for immunoblot analysis of p-IκB-α, NF-κB (p65) and actin by specified antibodies.

A

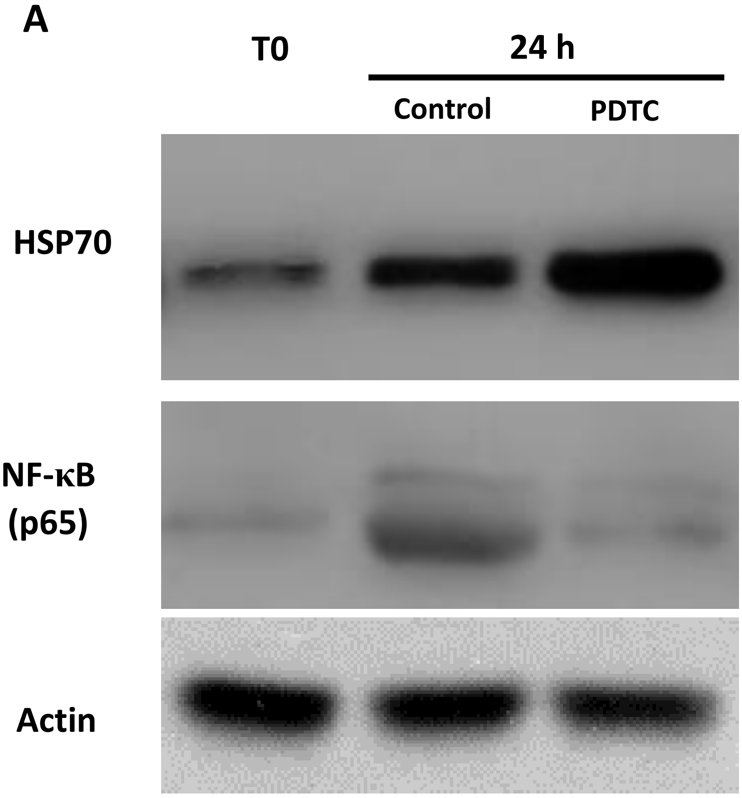
T0
24 h
Control
PDTC
HSP70
NF-κB
(p65)
Actin

B

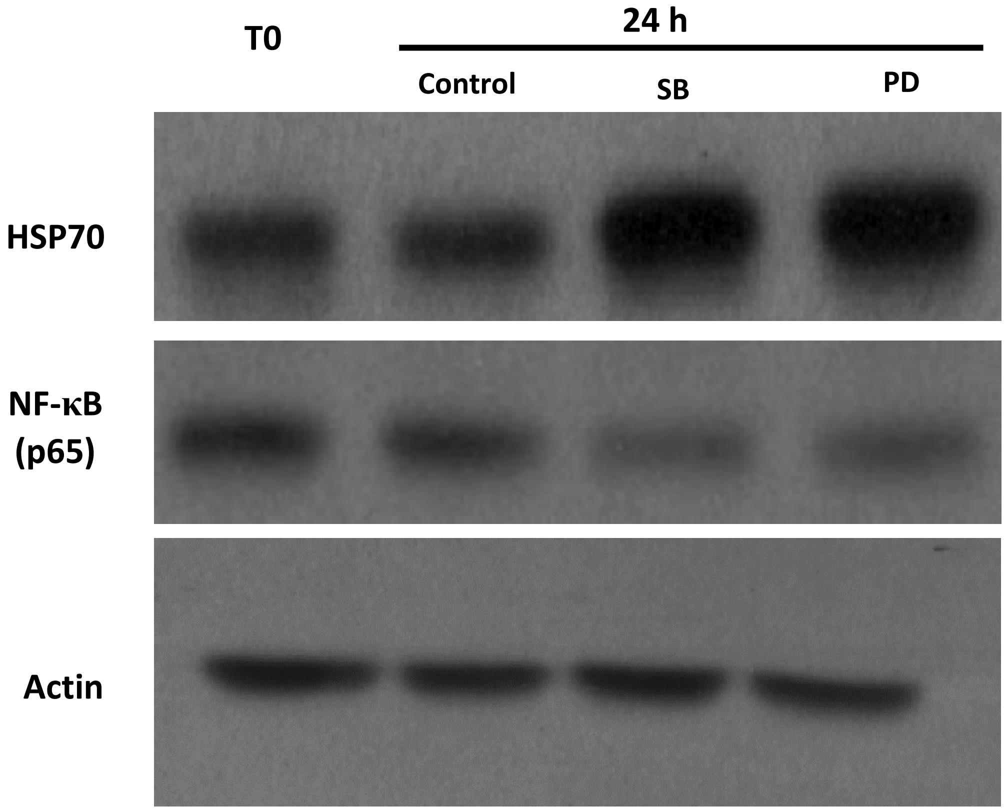
T0
24 h
Control
SB
PD
HSP70
NF-κB
(p65)
Actin

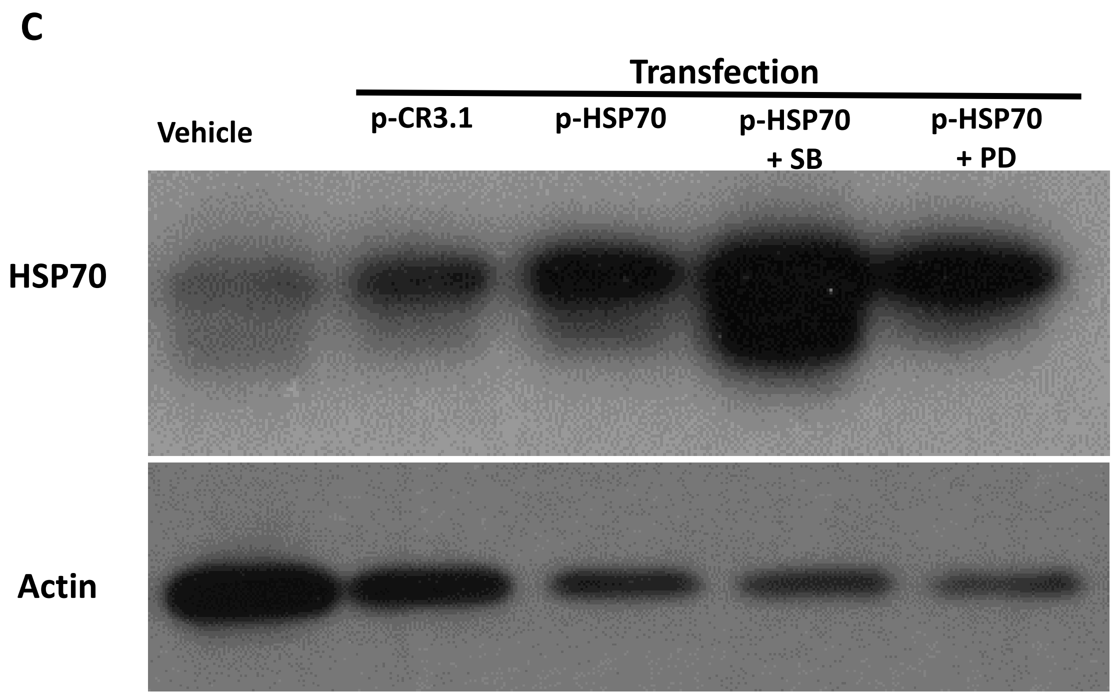

Supplementary Figure 3. Immunoblots of HSP70 or NF-κB (p65) in alveolar macrophages Alveolar macrophages derived from TB patient were treated in the presence or absence of (A) pyrrolidine dithiocarbamate (PDTC, an inhibitor of NF-κB), or (B) SB20358 (SB, a p38-MAPK inhibitor), or PD98059 (PD, an ERK inhibitor) for 24 hours. (C) Alveolar macrophages of TB patient were transfected with p-CR3.1 vector or p-CR3.1-HSP70 plasmid (p-HSP70), then incubated with medium alone (vehicle), in the absence or presence of a p38-MKAP inhibitor (p-HSP70+SB) and an ERK inhibitor (p-HSP70+PD) for 24 hours. The blots show the immunoblot analysis of HSP70, NF-κB (p65) and actin by using specified antibodies.

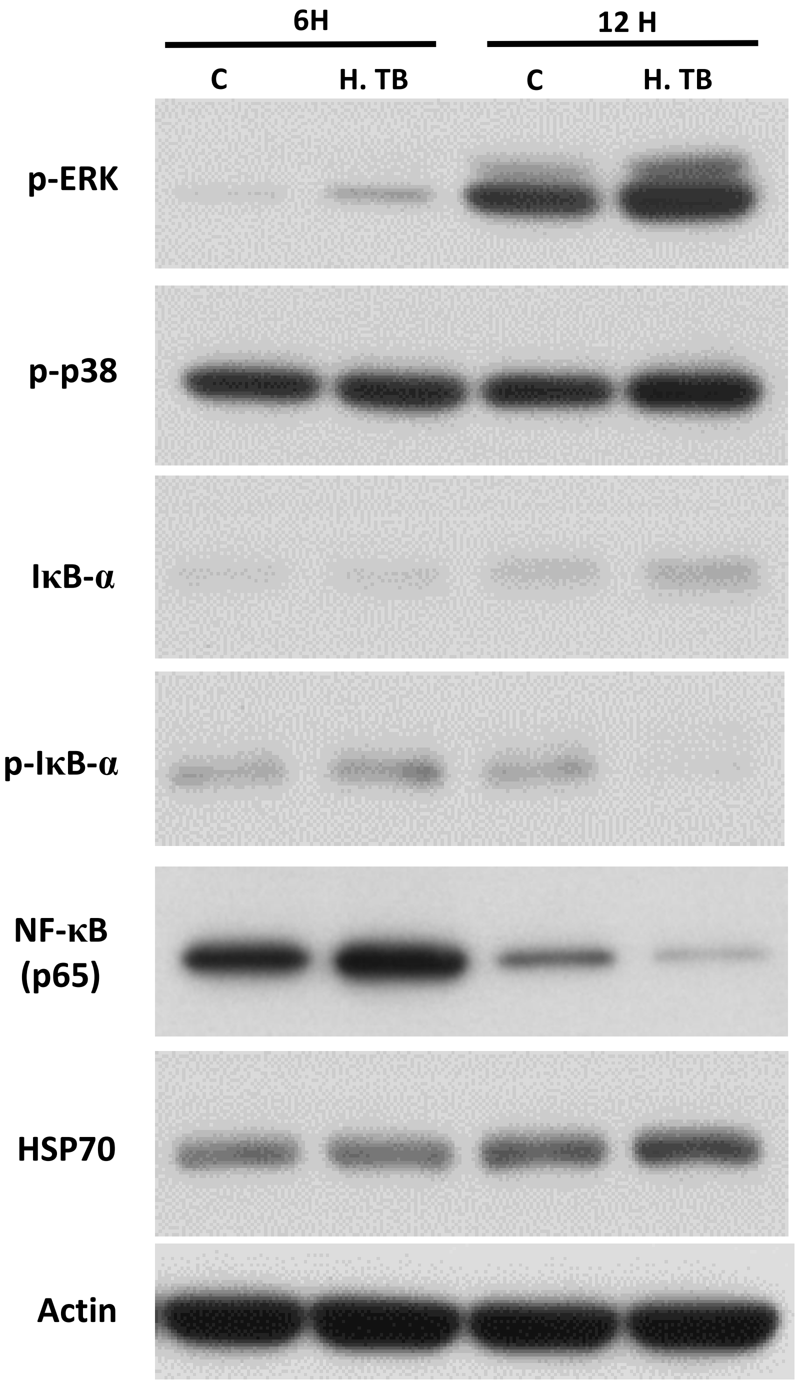

Supplementary Figure 4. Immunoblots of alveolar macrophages derived from normal subject in presence or absence of heated TB bacilli. The immunoblot analysis indicates the expression of MAPK (p38 and p-ERK), IκB-α, p-IκB-α, NF-κB p65, HSP70 and actin in alveolar macrophages of normal subject after incubation with heated TB bacilli for 6 and 12 hours.
